# Over-the-scope pre-looping method using an endoloop in endoscopic full-thickness resection of gastric gastrointestinal stromal tumor

**DOI:** 10.1055/a-2462-1333

**Published:** 2024-11-26

**Authors:** Reona Kawamura, Seiichiro Abe, Yasuhiko Mizuguchi, Satoru Nonaka, Yutaka Saito

**Affiliations:** 1 68380 Endoscopy Division, National Cancer Center Hospital, Chuo-ku, Tokyo, Japan

Endoscopic full-thickness resection (EFTR) for small gastric gastrointestinal stromal tumors (GISTs) is a feasible and effective procedure 1 . However, visual field obstruction during defect approximation post-EFTR often poses a challenge due to inadequate gastric distension 2 .

To address this, we developed a method where an endoloop is maneuvered over the scope to the circumferential incision, forming a “pre-loop” that allows for rapid approximation of the full-thickness defect after EFTR. A 72-year-old man presented with a submucosal tumor measuring 25 mm in diameter, suspected to be a GIST located on the anterior wall of the middle gastric body ( Fig. 1 ). The EFTR was performed in an operating room setting ( Video 1 ). To minimize the size of the full-thickness defect, submucosal dissection was initially performed around the lesion ( Fig. 2 ). After identifying the lesion’s capsule, an endoloop was advanced while grasping the scope tip and positioned over the scope ( Fig. 3 ). The loop was then opened at the mucosal incision site and anchored with endoclips, a technique we term “pre-looping” ( Fig. 4 ).

**Fig. 1:**
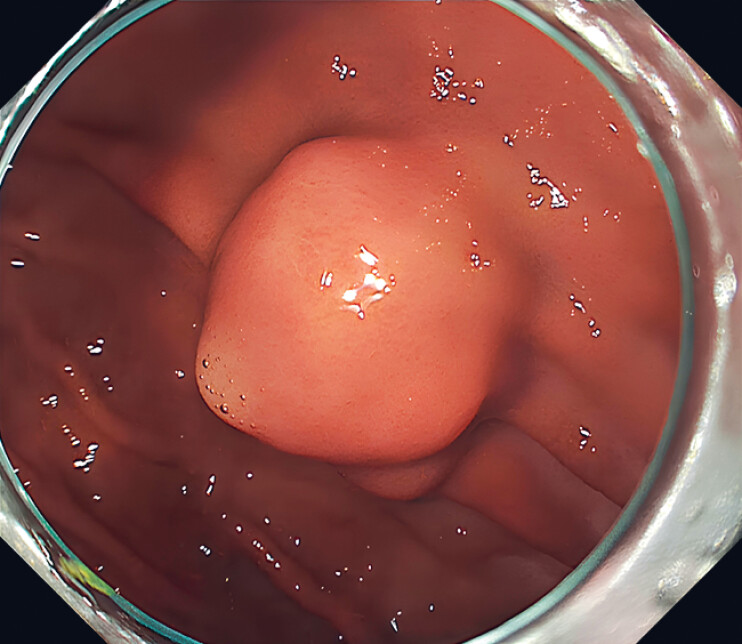
A 25-mm submucosal tumor located on the anterior wall of the middle part of the gastric body.

**Fig. 2:**
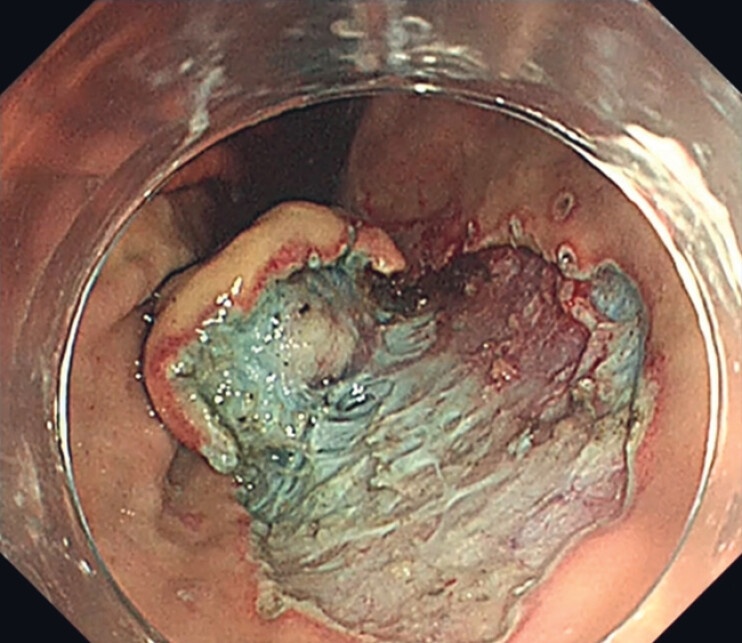
Submucosal dissection was performed to reduce the full-thickness defect area.

**Fig. 3:**
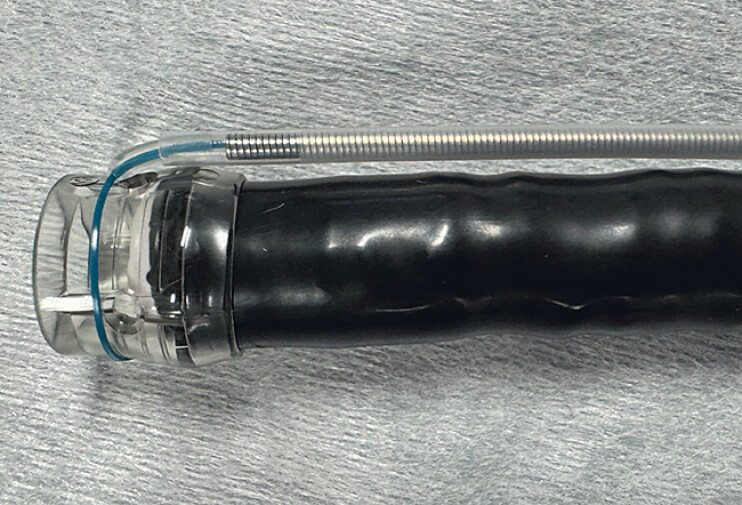
An endoloop was used to grasp the tip of the scope and placed over the scope.

**Fig. 4:**
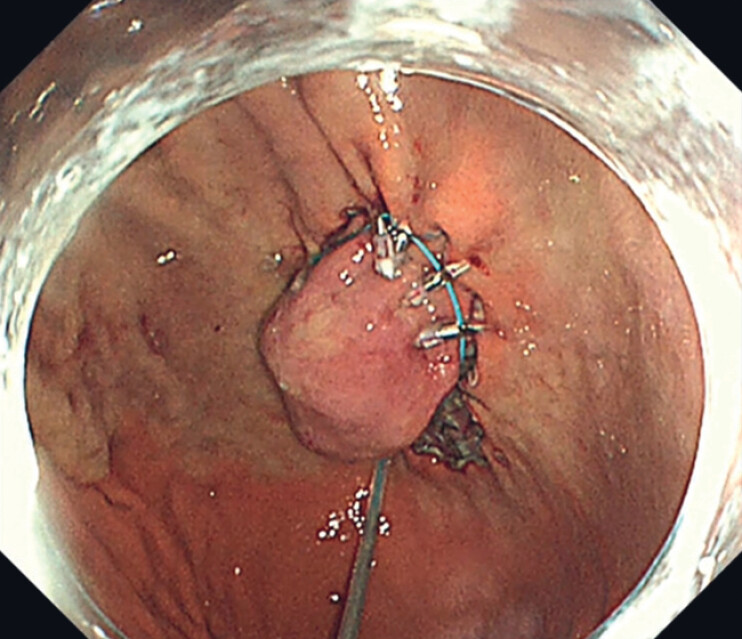
The endoloop was anchored using endoclips, a technique we term the “pre-looping” method.

Endoscopic full-thickness resection (EFTR) was performed in the operating room for a gastric gastrointestinal stromal tumor (GIST) in a 72-year-old man. An over-the-scope pre-looping suture method using an endoloop was performed after EFTR.Video 1

The procedure took 10 min, with endoloop approximation completed post-EFTR. Approximation of the full-thickness defect required an additional 3 min, followed by closure of a residual mucosal defect using a second endoloop ( Fig. 5 ). The total approximation time was 21 min.

**Fig. 5:**
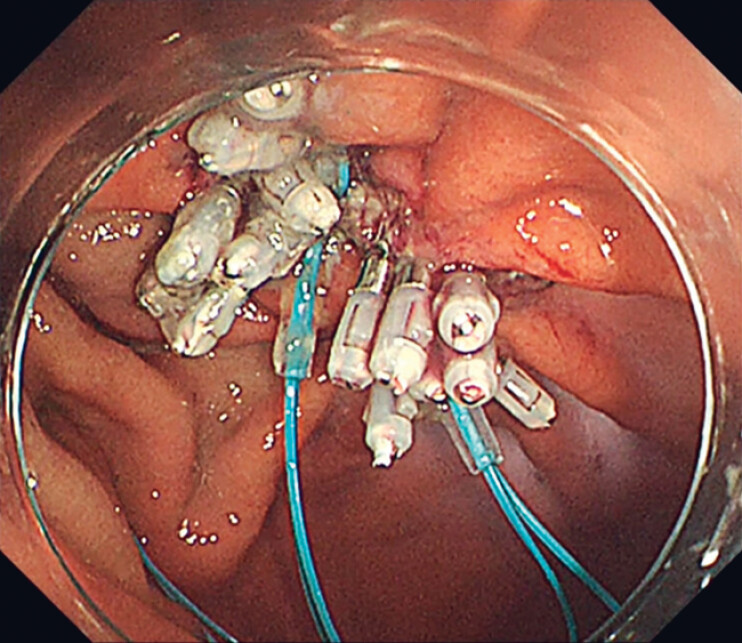
Complete approximation of the defect area following tumor excision.

The patient experienced no intraoperative or postoperative complications and was discharged on the fifth postoperative day. Pathological analysis confirmed a low-risk GIST with negative horizontal and vertical margins.

This suture technique remains effective even in cases where gastric lumen collapse follows EFTR, making it a quick and reliable method for defect closure. We intend to adopt this technique as a standard for EFTR in our hospital.

Endoscopy_UCTN_Code_TTT_1AO_2AG_3AF

## Notes

Endoscopy E-Videos https://eref.thieme.de/e-videos
*E-Videos* is an open access online section of the journal *Endoscopy* , reporting on interesting cases and new techniques in gastroenterological endoscopy. All papers include a high-quality video and are published with a Creative Commons CC-BY license. Endoscopy E-Videos qualify for HINARI discounts and waivers and eligibility is automatically checked during the submission process. We grant 100% waivers to articles whose corresponding authors are based in Group A countries and 50% waivers to those who are based in Group B countries as classified by Research4Life (see: https://www.research4life.org/access/eligibility/ ). This section has its own submission website at https://mc.manuscriptcentral.com/e-videos .
